# Impact of Ag/ZnO Reinforcements on the Anticancer and Biological Performances of CA@Ag/ZnO Nanocomposite Materials

**DOI:** 10.3390/molecules28031290

**Published:** 2023-01-29

**Authors:** Nadiyah Alahmadi, Mahmoud A. Hussein

**Affiliations:** 1 Department of Chemistry, College of Science, University of Jeddah, Jeddah 21959, Saudi Arabia; 2 Department of Chemistry, Faculty of Science, King Abdulaziz University, Jeddah 21589, Saudi Arabia; 3 Chemistry Department, Facul1ty of Science, Assiut University, Assiut 71516, Egypt

**Keywords:** cellulose acetate, Ag-doped ZnO, nanocomposite materials, green synthesis, antibacterial activity, anticancer activity

## Abstract

In this study, an unpretentious, non-toxic, and cost-effective dissolution casting method was utilized to synthesize a group of anticancer and biologically active hybrid nanocomposite materials containing biopolymer cellulose acetate. Pristine ZnO and Ag_(0.01, 0.05, 0.1)_/ZnO hybrid nanofillers based on variable Ag NP loadings were prepared via green procedures in the presence of gum arabic (GA). The chemical structures and the morphological features of the designed nanocomposite materials were investigated by PXRD, TEM, SEM, FTIR, TGA, and XPS characterization techniques. The characterization techniques confirmed the formation of CA@Ag_(0.01, 0.05, 0.1)_/ZnO hybrid nanocomposite materials with an average crystallite size of 15 nm. All investigated materials showed two degradation steps. The thermal stability of the fabricated samples was ranked in the following order: CA/ZnO < CA@Ag_(0.01)_/ZnO < CA@Ag_(0.05)_/ZnO = CA@Ag_(0.1)_/ZnO. Hence, the higher Ag doping level slightly enhanced the thermal stability. The developed nanocomposites were tested against six pathogens and were used as the target material to reduce the number of cancer cells. The presence of Ag NPs had a positive impact on the biological and the anticancer activities of the CA-reinforced Ag/ZnO composite materials. The CA@Ag_(0.1)_/ZnO hybrid nanocomposite membrane had the highest antimicrobial activity in comparison to the other fabricated materials. Furthermore, the developed CA@Ag_(0.1)_/ZnO hybrid nanocomposite material effectively induced cell death in breast cancer.

## 1. Introduction 

Owing to their inherently advantageous structural, electrical, and mechanical characteristics, polymer nanocomposites have experienced rapid growth in popularity and advancement over the past few generations. The use of nanofiller in a polymer host could be beneficial for a number of purposed, such as in biosensors, energy storage devices, photocatalysts, drug delivery, and other applications [1]. Biopolymers differ from the traditional polymers. They are created or obtained from living creatures, such as plants and microbes [2]. Biopolymers may be natural or synthetic in nature [1]. The use of biopolymers could produce material with unique properties, including biodegradability, biocompatibility, and sustainability [3]. Biodegradable polymers, or biopolymers, are synthetic materials that can be decomposed by microorganisms such as bacteria and fungi [1,3]. As a result, they do not harm the environment in any way [1]. Under aerobic conditions, biopolymers degrade into CO_2_, H_2_O, and biomass, whereas under anaerobic conditions, they decompose into methane, hydrocarbons, and biomass [3]. Therefore, biopolymers are promising substitutes for petroleum-based materials. Biopolymers can be categorized into three groups depending on their sources. Biomass biopolymers, such as polysaccharides, proteins, and lipids, are extracted from biomass. Biopolymers such as polylactic acid (PLA) can be chemically synthesized from biomass. Microorganism biopolymers, such as microbial polysaccharides and microbial polyesters, are produced by microorganisms [3]. Improvements in their mechanical and thermal characters can be achieved through nanoparticle inclusion into the polymeric matrices. A number of nanoparticles, including metallic-based materials and their oxides, as well as nanoclays, have been utilized to enhance the properties of polymers [4,5]. The development of biopolymers containing nanocomposites and the many fields to which they have been applied have made possible new avenues of study. Additionally, these types of nanocomposites are an emerging category of biohybrid composites that typically combine biopolymer matrices with nanoscale reinforcing elements [6]. Such eco-friendly NCs are well-rounded, so they should improve compatibility, recycling, and output rates [3]. Moreover, biopolymers containing nanocomposites have a highly flexible range of significant industrial uses in modern technology, such as for automotive parts, environmentally sound packaging utilities, biomedical applications, smart electronics, and a wide range of other applications [6].

Zinc oxide nanoparticles (ZnO NPs) constitute a multifunctional metal oxide because of their unique electronic structure. They are also classified as semiconductor materials (n-type), owing to the wide range of their bandgap (3.37 eV). Moreover, as non-toxic materials, they have potential in environmental and biological applications. ZnO NPs are applied in a wide range of applications, for instance, gas sensors, optoelectronic devices, dye-sensitized solar cells, and photocatalysts [7,8]. ZnO NPs have been incorporated into biopolymers for target applications. Althomali et al. modified glassy carbon electrodes with polyaniline@dialdehyde carboxymethyl cellulose/ZnO nanocomposites (PANI/D-CMC/ZnO) for the detection of H_2_O_2_ [9]. Akshaykranth et al. fabricated a novel nanocomposite that consisted of polylactic acid (PLA) and curcumin–ZnO to investigate its optical and antibacterial properties [10]. Kotharangannagari et al. applied hybrid nanocomposites of starch/lysine@ZnO NPs in food packing applications [5]. Because of their antibacterial properties, a lot of attention has been paid to silver nanoparticles (Ag NPs). They are widely used in biological applications, such as biosensors, forensic science, burn treatment, wound healing, biomolecule diagnostics, and chemotherapeutic processes [11,12,13,14]. It has been demonstrated that well-known oxide materials can be combined with biopolymers such as polysaccharides and their derivatives to form nanocomposites that either have a novel functionality or improve upon an existing function. Ail et al. used Ag/ZnO/chitosan (Ag/ZnO/Cs) ternary bionanocomposites to improve the antibacterial, optical, and photocatalytic properties of the metals [7]. Zare et al. combined Ag/Zn NPs with blended poly(3-hydroxybutyrate-co-3-hydroxyvalerate)-chitosan (PHBVCS) biopolymers to make food packaging that promotes longer shelf life [15]. Trandafilović et al. investigated the photocatalytic and antimicrobial activity of alginate-ZnO/Ag nanocomposites [16]. Shi et al. constructed Ag–ZnO/cellulose nanocomposites as effective photocatalysts for the photodegradation of methyl orange (MO) [17]. Peng et al. reported decorating cellulose/chitosan with Ag/Ag_2_O/ZnO (AZ@CC) to explore the resulting photocatalytic and antimicrobial activity [18]. Cellulose is the natural biopolymer that is found in the greatest abundance on the planet [17]. Among the most significant cellulose derivatives is cellulose acetate (CA) [19], which is extracted and synthesized from renewable and natural resources [20,21]. CA has attracted considerable attention, as it is a biodegradable polymer with hydrophilic features, excellent chemical and mechanical stability, and low toxicity [22,23]. Because of its advantages, CA has the potential to be broadly utilized in industrial and biomedical applications, including drug delivery, wound dressing, and separation membrane technology [24,25,26]. The number of studies related to CA have increased [27]. In addition, gum arabic is one of the cellulose derivatives that could be applied as a stabilizer in the synthesis of nanoparticles, as it is commercially available at low cost [28,29,30,31,32,33,34]. The main strategy employed in the present work was to estimate the anticancer and biological performances of cellulose acetate biopolymer membrane-reinforced Ag/ZnO hybrid nanomaterials. A green synthesis procedure was utilized to prepare the Ag/ZnO hybrid reinforcement agent in the presence of gum arabic. We also focused on structural investigations of the developed hybrid nanocomposite materials combined with variations in Ag/ZnO dopant concentrations referring to the silver loadings using PXRD, FTIR, TEM, SEM, XPS, and TGA techniques; morphological studies were also undertaken.

## 2. Experimental 

### 2.1. Reagents and Materials 

Silver nitrate (purity > 99%), zinc (II) nitrate hexahydrate (purity > 98%), cellulose acetate, sodium hydroxide pellets, ethanol, and acetone (analytical grade) were obtained from Sigma-Aldrich, Fisher Chemical, and Techno PharmChem India, respectively. Gum arabic (GA) was available from a commercial market. Deionized water was used to prepare all aqueous solutions. All chemicals were used as obtained. 

### 2.2. Preparation of Pristine ZnO Nanoparticles

Freen synthesis of pristine ZnO NPs was carried out as follows: an aqueous solution of Zn^2+^ (50 mL, 0.1 M) and GA (40 mL, 1% (*w*/*v*)) was stirred for 30 min, followed by an adjustment of the pH to 10. The stirring was continued for an additional 180 min. For a period of 24 h, the suspended solution was aged at room temperature. Before being dried in a furnace, the emulsion was first centrifuged; then, any residue that remained was thoroughly cleaned with ethanol and deionized water. The ZnO nanoparticles were then subjected to a calcination process for 1 h at 400 °C in an oven.

### 2.3. Green Synthesis of Ag_(0.01, 0.05, 0.1)_/ZnO Hybrid Nanomaterials

**Scheme 1:**
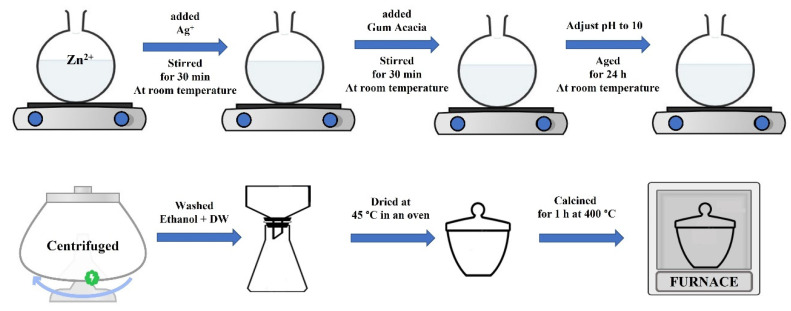
An illustration of the green synthesis of Ag_(0.01, 0.05, 0.1)_/ZnO hybrid nanomaterials.

A green synthesis for the Ag/ZnO hybrid nanomaterials based on variable Ag loading was carried out as follows. Equal amounts of Ag^+^ and Zn^2+^ and 40 mL of aqueous solution GA (1% (*w*/*v*)) were stirred together for 30 min. The pH value was adjusted to pH = 10, and the aqueous solution was left at room temperature for 24 h. After the colloidal was centrifuged, it was washed with a small amount of ethanol and deionized water. The suspended solids were then centrifuged again. The precipitate was then dried in the oven and subjected to a calcination process for 1 h at 400 °C in a furnace. The concentration of Zn^2+^ was kept constant at 0.1 M, and three Ag^+^ concentrations were used, i.e., 0.01, 0.05, and 0.1 M. Scheme 1 is an illustration of the preparation of Ag/ZnO hybrid nanomaterials.

### 2.4. CA/ZnO and CA@Ag/ZnO Hybrid Membrane Fabrication Procedures

The dissolution casting technique was utilized to fabricate the developed CA/ZnO and CA@Ag/ZnO hybrid membranes. A solution of 25 mL of acetone consisting of dissolved cellulose acetate powder (1 g) and a 10% fixed loading of pure ZnO nanoparticles was used to prepare the CA/ZnO hybrid membrane. The solution was stirred for 2 h, then sonicated for 30 min. The casting procedure was then carried out, and the homogeneous solution was poured into a glass petri dish. To avoid contamination from environmental particles, the dishes were wrapped in aluminum foil and dried for 24 h. Similar procedures were applied for the fabrication of the CA@Ag_(0.01–0.1)_/ZnO hybrid membranes using three different loadings of Ag (0.01, 0.05, and 0.1) each time. 

### 2.5. Utilized Instrumentation

The properties of the CA biopolymer, the prepared green nanomaterials, and the developed CA/ZnO and CA@Ag/ZnO hybrid membranes were studied using a number of characterization techniques, such as PXRD, FTIR, TEM, SEM, TGA, and XPS. The instrumentation used in this study was as follows. The PXRD pattern from 5° to 80° was recorded with a Bruker D8 Advance X-ray diffractometer using Cu K (=1.5406A) radiation at 40 kV and 20 mA. The FTIR spectra were recorded by (FT/IR-4100, JASCO, Japan) in the range 400–4000 cm^−1^. The produced hybrid materials were analyzed using a TGA-50 Shimadzu Thermo gravimetric analyzer. We investigated the morphology and chemical makeup using a JEMF200 multipurpose electron microscope and a JSM-7610F Plus Schottky field emission scanning electron microscope. X-ray photoelectron spectra were captured using a Thermo Scientific K-Alpha™ spectrometer (XPS).

### 2.6. Biological Screening

The biological activities of CA biopolymer and the developed CA/ZnO and CA@Ag/ZnO hybrid membranes were displayed via the agar diffusion technique. They were tested against a wide range of bacterial species, Gram-negative bacteria strains, Gram-positive bacteria strains, and fungi; for example, Serratia marcescens ATCC 21074 and Escherichia coli ATCC 35218; Bacillus Cereus ATCC 14579 and Staphylococcus aureus ATCC 29213; and Candida albicans ATCC 76615 and Aspergillus flavus ATCC 9643. All microorganisms were supplied by King Abdulaziz University (microbiology lab located at King Fahad Medical Center) in Jeddah, Saudi Arabia. Previous studies revealed the methodology used to evaluate antibacterial efficacy of the new nanocomposites [35]. Table 1 displays the results of measuring the size of the growth inhibition zone.

### 2.7. In Vitro MCF7 Anticancer Activity

#### 2.7.1. Cell Culturing

The breast cancer cell lines (MCF-7) were cultured in Dulbecco’s Modified Eagle Medium (DMEM), which was complemented with 10% fetal bovine serum (FBS), 100 g/mL streptomycin, and 100 units/mL penicillin. After the cell lines had developed to their full potential, they were subjected to an incubation period at a temperature of 40 degrees Celsius. The compounds that were analyzed were suspected of having undergone many doses of testing with MCF-7.

#### 2.7.2. Experimental (Cell Count and Cell Viability) Investigations

The CA@Ag_(0.1)_/ZnO nanocomposite was chosen for this investigation because it had previously been explored for its antimicrobial activity. In the previous investigation, it was shown to demonstrate a modest level of antimicrobial activity against the bacteria and fungus that were being studied. Over the course of 48 h, MCF-7 breast cancer cell lines were cultivated in two different environments: in the absence of CA@Ag_(0.1)_/ZnO nanocomposite and in its presence at several concentrations (0, 0.5, 1, 2, and 3 mg/mL). Following the incubation time, microscopic pictures were captured using inverted microscopy. Thereafter, MCF-7 cell lines were harvested and quantified utilizing a hemocytometer [36,37,38]. 

In addition, an MTT test was carried out to determine cell viability according to the recommendations provided by the manufacturer (Invitrogen, Carlsbad, CA, USA) [39]. This test assessed the viability of cells by evaluating the metabolic activity of the cells that were found to be viable. In this regard, MCF-7 cell lines were implanted in 96-well, flat-bottomed plates, and cells were plated in the 96-multiwell plate (104 cells/well) for 24 h prior to treatment with the material to enable cell adhesion to the plate wall. Experiments were carried out for forty-eight hours, first in the absence of the CA@Ag_(0.1)_/ZnO nanocomposite and then in its presence at varied concentrations, namely 0, 0.5, 1, 2, and 3 mg/mL.

## 3. Results and Discussion 

### 3.1. Chemical Structure Evaluations of CA/ZnO and CA@Ag/ZnO Hybrid Membranes

The chemical structure of the developed CA/ZnO, as well as that of CA@Ag/ZnO in hybrid membranes with variable silver loadings, was evaluated by eco-friendly and cost-effective dissolution casting methods. Prior to the fabrication process, a green preparation of pristine ZnO and hybrid Ag-doped ZnO NPs was carried out in the presence of gum arabic. The solution’s immediate color change from colorless to dark gray confirmed the reduction in silver ions. GA is a water-soluble polysaccharide-based biomaterial. The advantage of gum arabic in this application is its ability to stabilize the desired hybrid nanocomposite materials.

**Figure 1:**
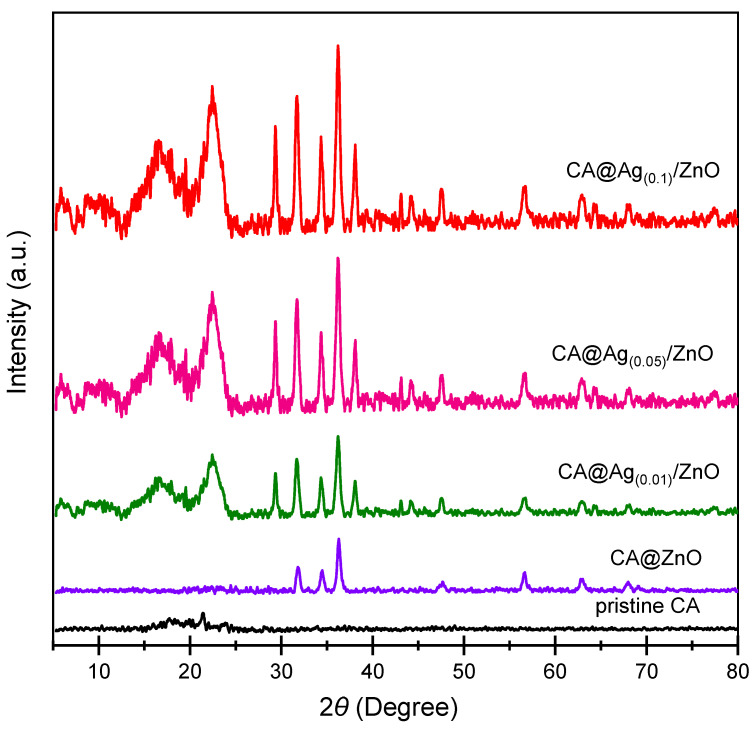
PXRD diffractograms of pristine CA, CA/ZnO, and CA@Ag_(0.01, 0.05, 0.1)_/ZnO hybrid membranes.

The PXRD features of CA, CA/ZnO, and CA@Ag_(0.01–0.1)_/ZnO hybrid composite membranes are shown in Figure 1. This figure shows that pristine CA had a typical diffraction pattern, including two separate diffraction peaks at 18 and 22° in the 2 direction, as previously reported in the literature [40]. The crystalline nature of CA@ZnO was demonstrated by a hexagonal structure with a P 63 mc space group, which is consistent with the reference data for the material (JCPDS no. 36-1451). CA@Ag_(0.01, 0.05, 0.1)_/ZnO hybrid nanocomposites had a face-centered cubic crystalline structure, as reflected by their respective PXRD patterns, i.e., space group Fm-3m. These peaks were well-matched with the reference peaks of silver (JCPDS no. 04-0783). The refraction peaks at 32.79 and 54.9° indicated the existence of Ag NPs. Figure 1 confirms the doping of the ZnO NPs with Ag NPs. Moreover, the ZnO NPs maintained their crystal structure. Scherrer’s formula was applied to the FWHM of the prominent peaks and their location to derive the crystallite size (D) of the produced nanocomposite (nm). The pristine CA crystallite size (D) was 3.1 nm, whereas the average crystallite size of the fabricated nanocomposite membranes was approximately 15 nm.

**Figure 2:**
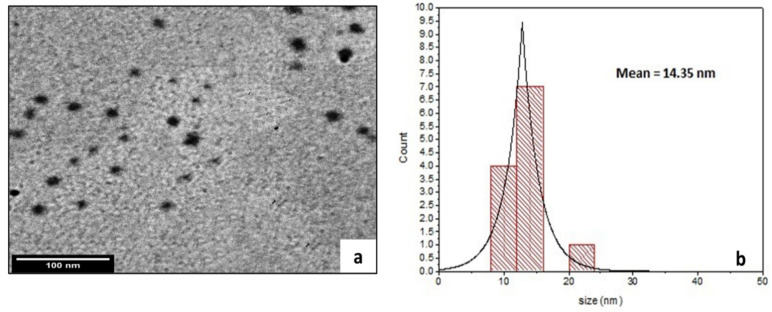
TEM image of CA@Ag_(0.1)_/ZnO hybrid membrane (**a**) and a histogram of its particle size distribution (**b**).

Figure 2 displays a TEM image of CA@Ag_(0.1)_/ZnO hybrid membrane (a) as a selected example, and its related particle size distribution histogram is shown in Figure 2b. It can be seen from the image that the prepared sample had a spherical shape, and the average size of nanoparticles was 15 nm. The Ag/ZnO hybrid showed improved compatibility and good distribution of nanoparticles into the CA polymer matrix.

**Figure 3:**
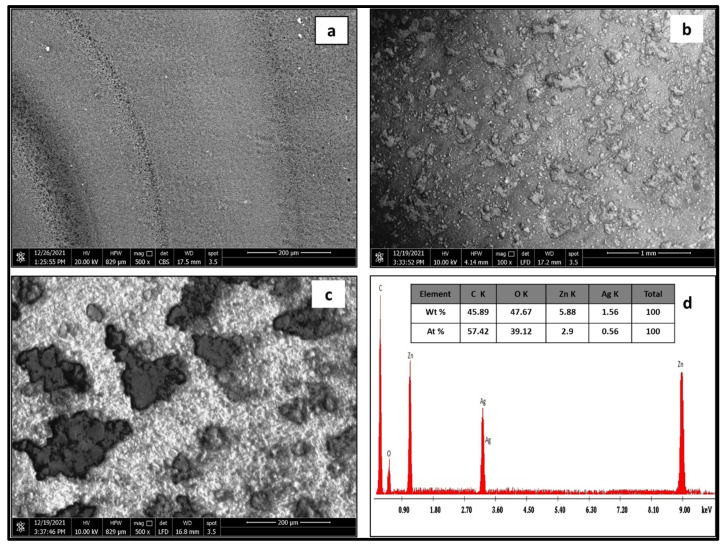
SEM micrographs of pristine CA (**a**) CA@Ag_(0.1)_/ZnO hybrid material at low (**b**) and high magnifications of (**c**) and EDX analysis of CA@Ag_(0.1)_/ZnO hybrid material (**d**).

Figure 3 displays SEM micrographs of pristine CA (a) CA@Ag_(0.1)_/ZnO hybrid material (b), a high-resolution image of the CA@Ag_(0.1)_/ZnO hybrid material (c), and the EDX signals and their percentage composition of the CA@Ag_(0.1)_/ZnO hybrid material (d). Figure 3c shows that after the surface of CA was changed with a Ag_(0.1)_/ZnO hybrid membrane, a variety of accumulated bubbles of different shapes appeared. Energy-dispersive X-ray (EDX) was used to examine the elemental composition of the prepared sample, as seen in Figure 3d. There were two peaks at 1.2 and 9.00 keV denoting Zn. The peak at 0.5 keV denoted O. The peak at 3.0 keV corresponded to silver. Moreover, an energy peak of C linked to carbon in CA was also present. These findings confirm the formation of a CA@Ag_(0.1)_/ZnO hybrid nanocomposite membrane. 

**Figure 4:**
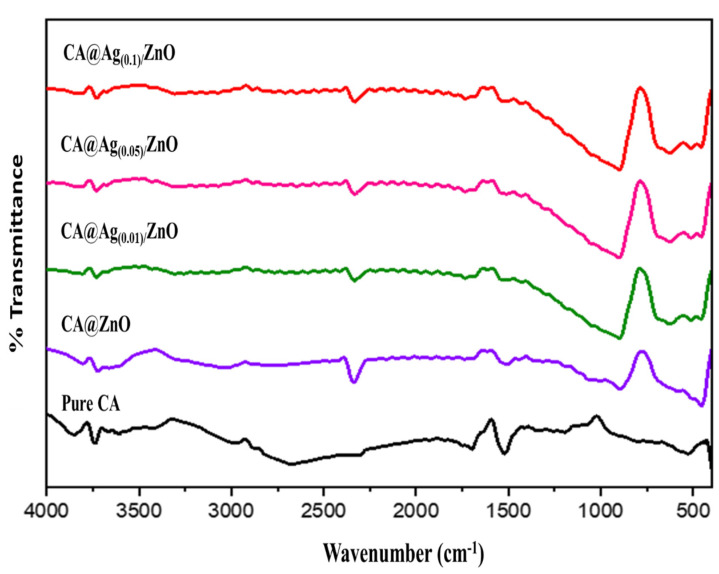
FTIR of pristine CA, CA/ZnO, and CA@Ag_(0.01, 0.05, 0.1)_/ZnO hybrid materials.

Figure 4 shows the FTIR analyses of the developed hybrid materials in the range of 500–4000 cm^−1^. FTIR analysis clarified the material functionality and nanocomposite formation of the hybrid nanocomposite. Figure 4 shows the FTIR spectra of pristine CA (black line), CA/ZnO composite material (purple line), and CA@Ag_(0.01, 0.05, 0.1)_/ZnO hybrid materials as final targeted products (green, pink, and red lines, respectively). The FTIR spectrum of pure CA showed an absorption-stretching broadband around 3400 cm^−1^, representing the presence of hydroxyl groups. The C-H group was obtained from the foundations of various peaks centered at 2973 and 1373 cm^−1^. The peak at 1061 cm^−1^ was attributed to the C-O group. The presence of the peak around 1500 cm^−1^ was attributed to the stretching of the CA composition of the C=O ester carbonyl group. The peak at 1061 cm^−1^ was attributed to the 1120 cm^−1^ (acetate C-C-O stretching) and 1016 cm^−1^ (C-O stretching), which are characteristic of CA [41,42]. The FTIR spectra of CA@ZnO hybrid materials (purple line) display a characteristic peak of CA. In addition, a new peak around 477 cm^−1^ was assigned to ZnO vibration, which further confirmed the formation of ZnO [42]. It can be seen that after the formation of of silver nanoparticles on the surface of the zinc oxide nanoparticles, the intensity of the ZnO peaks decreased in CA@Ag_(0.01, 0.05, 0.1)_/ZnO (green, pink, and red lines, respectively) [43].

**Figure 5:**
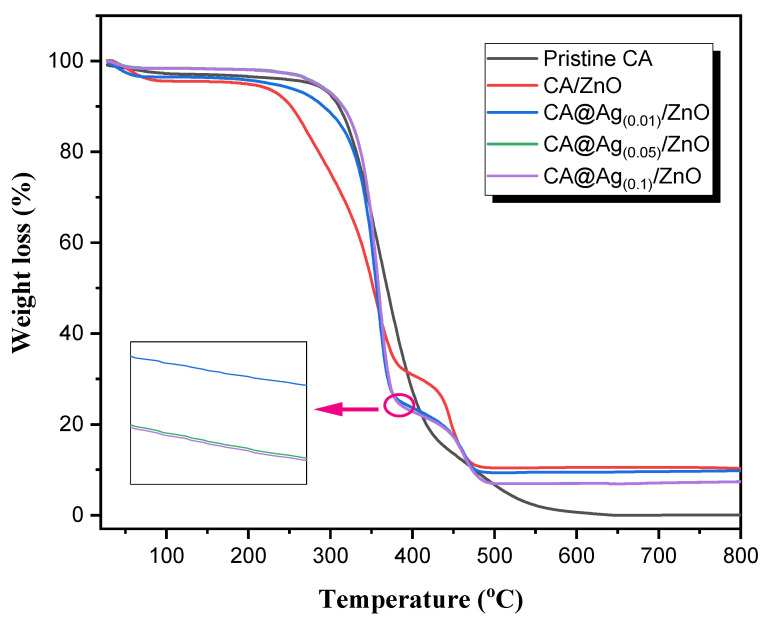
TGA thermograms of pristine CA, CA/ZnO and CA@Ag_(0.01, 0.05, 0.1)_/ZnO hybrid materials.

Figure 5 illustrates the TGA patterns of pristine CA, CA/ZnO, and CA@Ag_(0.01, 0.05, 0.1)_/ZnO hybrid materials. An initial weight loss amounting to ~10% occurred at around 100 °C in all samples as a result of the removal of H_2_O molecules and/or trapped solvents on the surface. The TGA thermogram for the pristine CA (black line) displayed single-step weight losses. This TGA thermogram also exhibited a sharp reduction in weight; 50% of the weight loss was observed at temperatures less than 400 °C. The thermal degradation of pristine CA was complete at 600 °C. In the fabricated CA/ZnO and CA@Ag_(0.01,0.05, 0.1)_/ZnO hybrid materials, the TGA curves mainly displayed two-step weight losses. The CA/ZnO thermogram showed lower thermal degradation in the first step compared to the CA, which meant that the addition of ZnO accelerated the thermal decomposition of the pristine CA. Meanwhile, it displayed a significant increase in thermal stability in the second step. The first step was rapid, starting at around 217 °C and ending at around 385 °C, whereas the second step was slow and was completed at around 492 °C. Furthermore, CA@Ag_(0.01,0.05, 0.1)_/ZnO hybrid materials showed identical decomposition patterns consisting of two main decomposition stages. The TGA curves of CA@Ag_(0.05)_/ZnO and CA@Ag_(0.1)_/ZnO were nearly identical (a tiny shift was observed, as illustrated in the subfigure of Figure 5). The first step was rapid, starting at around 264 °C and ending at around 385 °C. The second step was complete at around 500 °C. However, there was a noticeable shift in the TGA curve of CA@Ag_(0.01)_/ZnO. This shift was clearly noted between 250 and 350 °C. The first step was rapid, starting at 200 °C and ending at around 386°C. The second step was complete at around 425 °C. An almost 75% weight loss was observed in all the fabricated hybrid materials at around 450 °C. The thermal stability was highly affected by the silver loading in the fabricated hybrid materials. The thermal stabilities for those developed materials were detected in the following order: CA/ZnO < CA@Ag_(0.01)_/ZnO < CA@Ag_(0.05)_/ZnO = CA@Ag_(0.1)_/ZnO.

**Figure 6:**
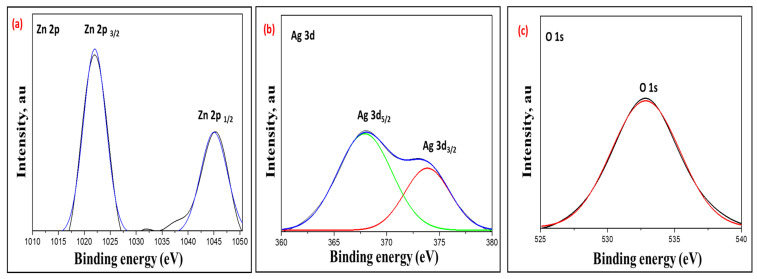
High-resolution XPS spectra of CA@Ag_(0.1)_/ZnO hybrid material, the Zn 2p (**a**), Ag 3d (**b**), and O1s (**c**).

Figure 6 displays the high-resolution XPS spectra of the fabricated CA@Ag_(0.1)_/ZnO hybrid nanocomposite. Figure 6a presents two observable peaks at 1045.00 eV and 1022.01 eV that are characteristic of Zn 2p_1/2_ and Zn 2p_3/2_, respectively. This observation confirms the presence of Zn^2+^ ions within the fabricated hybrid nanocomposite. Figure 6b presents the high-resolution Ag 3d spectrum giving rise to two peaks at 367.91 and 373.85 eV, corresponding to the Ag 3d_5/2_ and Ag 3d_3/2_ orbitals typical of Ag, respectively. Figure 6c shows a distinct peak realized at 532.84 eV, which could be correlated to O1s. This observation endorses the presence of an oxide lattice phase within the hybrid nanocomposite [44,45,46].

### 3.2. Antimicrobial Activities

**Figure 7:**
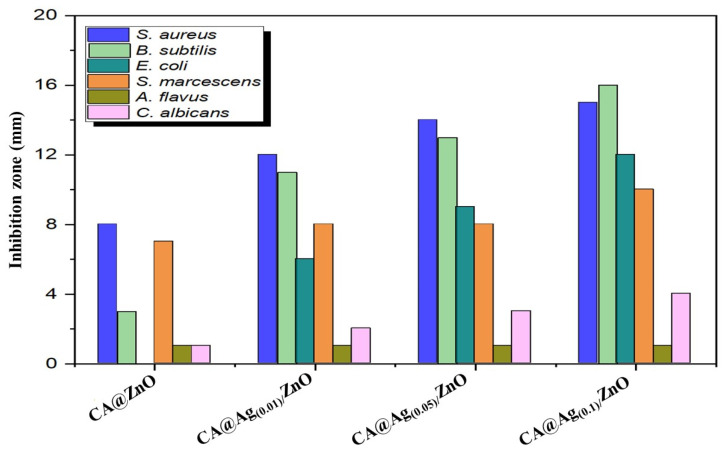
Antimicrobial activities of CA/ZnO and CA@Ag_(0.01, 0.05, 0.1)_/ZnO hybrid materials in the presence of selected microorganisms.

The fabricated CA/ZnO and CA@Ag_(0.01, 0.05, 0.1)_/ZnO hybrid materials were biologically screened against some selected bacterial and fungal microorganisms; the obtained results are illustrated in Table 1 and Figure 7. The biological characters showed that the presence of Ag NPs influenced the antibacterial capabilities of the developed nanocomposite materials. Table 1 records the effect of each tested material shown in the obtained inhibition zones (mm) of the bacterial species and fungi, whereas Figure 7 shows a screening illustration of CA/ZnO and CA@Ag_(0.01, 0.05, 0.1)_/ZnO hybrid materials against the same investigated microorganisms. A disk diffusion method was applied to evaluate the antimicrobial activity assay of prepared samples. It can be seen from Table 1 and Figure 7 that all fabricated hybrid composite materials demonstrated significant biological performance against the majority of the tested bacteria and fungi, except A. flavus. Moreover, there was a proportional relation between the concentration of Ag NPs in the prepared samples and the inhibition zone (mm). The replication process and the growth of microorganisms were noticeably affected by the increase in Ag NP loading. Thus, the CA@Ag_(0.1)_/ZnO hybrid nanocomposite membrane had the highest antimicrobial activity among the prepared samples. The biological properties showed that the presence of Ag NPs influenced the antibacterial capabilities of the nanocomposite.

Many different mechanisms have been proposed for the inhibition and destruction of bacterial cells [18,47,48]. According to some studies, electrostatic interaction between nanomaterials and microorganisms would release a positive ion that would lead to membrane breakdown or permeability disruption; for instance, ZnO NPs and Ag NPs would produce Zn^2+^ and Ag^+^ ions, respectively, which would lead to cell death [7]. Such ions may have the potential to interfere with the negatively charged functional groups (such as –NH, –COOH, and –SH) that are found in proteins and nucleic acids, which may lead to the suppression of DNA replication and the rupture of cell walls. Moreover, it has been reported in the literature that the formation of reactive oxidation species (ROS), such as hydroxyl radical (·OH) and hydrogen peroxide (H_2_O_2_), superoxide radical (O_2_^−^), and singlet oxygen (O_2_), can cause bacterial cell death. These species exhibited different levels of activity and dynamics with ZnO NPs and Ag NPs. ROS caused oxidative stress in bacteria, resulting in cell death. Moreover, the presence of Ag would reduce the recombination rate of the electron–hole pair, enhancing the production of ROS. It has been suggested that the cause of cell death may be the direct interaction between various NPs and the bacterial membrane [18,47,48].

### 3.3. In Vitro MCF7 Anticancer Activity

**Figure 8:**
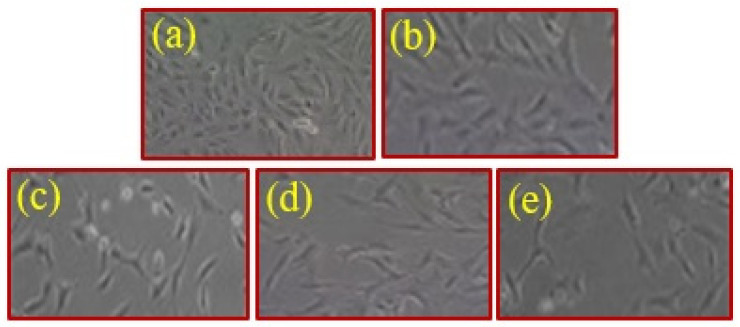
Images of MCF7 cells for the control (**a**) and in the presence of variable concentrations of CA@Ag_(0.1)_/ZnO nanocomposite 0.50, 1, 2, and of 3 mg/mL (**b**–**e**) at a magnification of X = 600.

Figure 8 shows images of the MCF7 cell used as a control (a) and that in the presence of variable concentrations of CA@Ag_(0.1)_/ZnO hybrid composite material (0.50, 1, 2, and 3 mg/mL) (b-e) at a magnification of X = 600. Figure 8 also shows the relationship between cellular uptake and the cytotoxicity of the hybrid nanocomposite. In addition, the results in Figure 8 indicate the positive effect of the hybrid nanocomposite on the health of cells as depicted by their more flattened appearance. Because of this effect, the CA@Ag_(0.1)_/ZnO hybrid nanocomposite induces cell death in breast cancer. This result is consistent with the findings reported in the abovementioned figure. 

**Figure 9:**
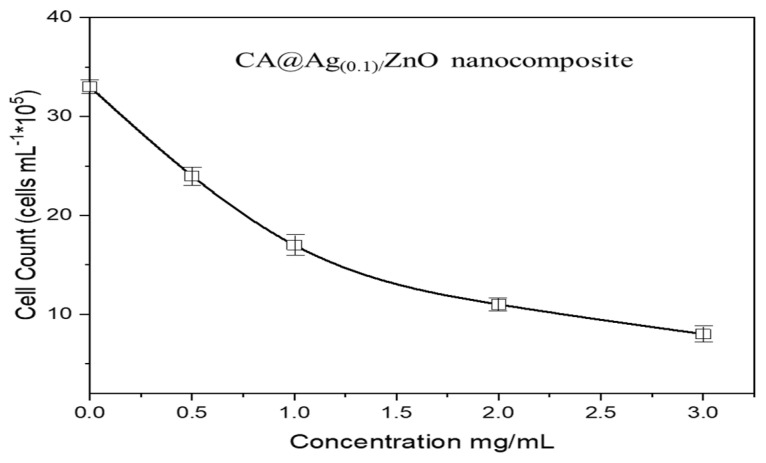
MCF7 cell counts at variable concentrations of CA@Ag_(0.1)_/ZnO hybrid material (0.50, 1, 2, and 3 mg/Ml).

**Figure 10:**
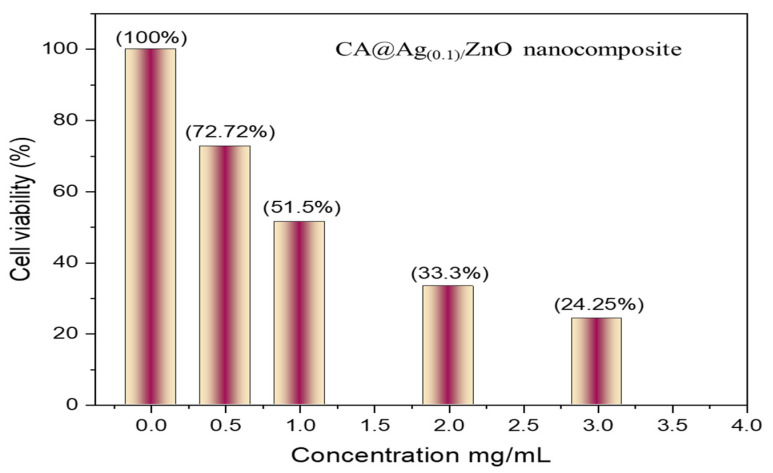
MCF7 cell viability at variable concentrations of CA@Ag_(0.1)_/ZnO hybrid material (0.50, 1, 2, and 3 mg/mL).

Furthermore, Figure 9 displays the MCF7 cell counts at variable concentrations of CA@Ag_(0.1)_/ZnO nanocomposites, namely 0.50, 1, 2, and 3 mg/mL. It can be concluded that the cytotoxicity potential associated with the nanocomposites occurred in a concentration-dependent manner. The hybrid nanocomposite caused 50% cell growth inhibition at a concentration of 1 mg/Ml. Moreover, Figure 10 displays the MCF7 cell viability at variable concentrations of CA@Ag_(0.1)_/ZnO hybrid material, namely 0.50, 1, 2, and 3 mg/mL. 

## 4. Conclusions

In order to manufacture a series of biologically active hybrid composite materials depending on cellulose acetate-reinforced hybrid Ag/ZnO nanomaterials, an easy, non-toxic, and cost-effective casting approach was efficiently utilized. A green synthesis method was used to prepare ZnO NPs and Ag-doped ZnO in three different concentrations. The chemical structure and morphologies of the developed composite materials were characterized using a number of techniques, such as PXRD, TEM, SEM, FTIR, TGA, and XPS. The average crystallite size (nm) for such hybrid materials was 15 nm. The doping of the ZnO NPs with Ag NPs and Ag_2_O NPs was confirmed. The hybrid nanocomposite membranes were spherical, exhibiting thermal stability at temperatures over 400 °C. The biological properties of fabricated materials were tested against selected bacterial and fungal species using a common biological tool. The results showed that the fabricated materials did not demonstrate any antimicrobial activities against *A. flavus*. The growth of the tested microorganisms was noticeably affected by the increase in the concentration of Ag NP loading. Moreover, hybrid nanocomposite membranes were used as target materials to reduce the number of MCF7 cancer cell lines. In addition, the cell count and cell viability in the presence of Ag NPs were analyzed through an MTT test. The biological properties showed that the presence of Ag NPs influenced the antibacterial capabilities of the nanocomposite.

## Figures and Tables

**Table 1:** Antimicrobial activities of CA/ZnO and CA@Ag_(0.01, 0.05, 0.1)_/ZnO hybrid materials.

Symbol	Microorganism Species/Inhibition Zone (mm)					
	*S. aureus*	*B. subtilis*	*E. coli*	*S. marcescens*	*A. flavus*	*C. albicans*
CA/ZnO	8	3	-	7	-	2
CA@Ag_(0.01)_/ZnO	12	11	6	8	-	4
CA@Ag_(0.05)_/ZnO	14	13	9	8	-	6
CA@Ag_(0.1)_/ZnO	15	16	12	10	-	9

## Data Availability

Not applicable.

## Sample Availability

Samples of the compounds are not available from the authors.
